# Predicting *Aedes aegypti* infestation using landscape and thermal features

**DOI:** 10.1038/s41598-020-78755-8

**Published:** 2020-12-10

**Authors:** Camila Lorenz, Marcia C. Castro, Patricia M. P. Trindade, Maurício L. Nogueira, Mariana de Oliveira Lage, José A. Quintanilha, Maisa C. Parra, Margareth R. Dibo, Eliane A. Fávaro, Marluci M. Guirado, Francisco Chiaravalloti-Neto

**Affiliations:** 1 Department of Epidemiology, School of Public Health - University of Sao Paulo, Av. Dr. Arnaldo, São Paulo, SP 715 Brazil; 2 Department of Global Health and Population, Harvard T.H. Chan School of Public Health, Boston, MA USA; 3 Southern Regional Centre of the National Institute for Space Research (INPE), Santa Maria, RS Brazil; 4 Virology Research Laboratory, Faculty of Medicine of São José do Rio Preto, São José do Rio Preto, SP Brazil; 5 Scientific Division of Management, Environmental Science and Technology of the Institute of Energy and Environment - IEE of University of Sao Paulo, São Paulo, SP Brazil; 6 Entomology Laboratory, Endemics Control Superintendence, São Paulo, SP Brazil; 7 Vectors Laboratory, Endemics Control Superintendence, São José do Rio Preto, SP Brazil

**Keywords:** Disease prevention, Health policy, Epidemiology

## Abstract

Identifying *Aedes aegypti* breeding hotspots in urban areas is crucial for the design of effective vector control strategies. Remote sensing techniques offer valuable tools for mapping habitat suitability. In this study, we evaluated the association between urban landscape, thermal features, and mosquito infestations. Entomological surveys were conducted between 2016 and 2019 in Vila Toninho, a neighborhood of São José do Rio Preto, São Paulo, Brazil, in which the numbers of adult female *Ae. aegypti* were recorded monthly and grouped by season for three years. We used data from 2016 to 2018 to build the model and data from summer of 2019 to validate it. WorldView-3 satellite images were used to extract land cover classes, and land surface temperature data were obtained using the Landsat-8 Thermal Infrared Sensor (TIRS). A multilevel negative binomial model was fitted to the data, which showed that the winter season has the greatest influence on decreases in mosquito abundance. Green areas and pavements were negatively associated, and a higher cover of asbestos roofs and exposed soil was positively associated with the presence of adult females. These features are related to socio-economic factors but also provide favorable breeding conditions for mosquitos. The application of remote sensing technologies has significant potential for optimizing vector control strategies, future mosquito suppression, and outbreak prediction.

## Introduction

Mosquitoes are responsible for the transmission of several infectious diseases, such as malaria, dengue, yellow fever, Zika virus, and filariasis and have become an increasing problem as a result of climate change, environmental changes, urban growth patterns, and insecticide resistance^1^. For example, the number of dengue cases reported to the World Health Organization (WHO) increased from < 0.5 million in 2000 to over 4 million in 2019^2^—the largest increase ever recorded. This included over 3.1 million cases in the Americas alone, of which more than 25,000 cases were classified as severe. For this reason, there have been significant attempts to improve surveillance methods for the quick detection and diagnosis of potential outbreaks of mosquito-borne diseases^3^. Notwithstanding mosquito monitoring policies have been developed worldwide^4–8^, monitoring in urban areas faces many challenges. In particular, for *Aedes aegypti*, the primary vector of dengue, chikungunya, and Zika virus^9^, control is laborious and inefficient given that the larvae prefer small, artificial habitats that are ubiquitous in urban areas. Surveillance efforts are also threatened by the wide variety of potential habitats as well as their ephemeral nature^10^. Therefore, despite some regional-scale efforts^7^, a crucial issue is being able to predict mosquito abundance without extensive and expensive fieldwork. Even though interventions exist, mosquito infestation and spread of arbovirus continue. The reasons for this include inadequate program implementation, ineffective coverage, and lack of human, financial, and infrastructural capacity^1^.

Climatic and landscape variables can be useful for predicting the local abundance and potential for expansion of arthropod vectors, including mosquitoes^11–14^. Given that field surveys are both costly and inefficient, remote sensing technologies are being increasingly used to estimate habitat suitability for a diversity of mosquito genera, including *Anopheles*^12,15^ and *Aedes*^5,14,16,17^. Specific land use and cover types can favor the proliferation of mosquitoes. For example, mosquito abundance and the occurrence of particular species is strongly influenced by vegetation cover and turbidity within standing bodies of water^18–21^. Lorenz et al.^22^ recently showed that infestations of *Ae. aegypti* adult mosquitoes were positively associated with the presence of asbestos roofing and roof slabs in an urban region of Brazil. In our study, we analyzed the same area studied by Lorenz et al.^22^ and used similar methodology, but we used a bigger dataset (all seasons for 3 years) and included climatic information (thermal images and precipitation). Furthermore, temperature and rainfall are climate parameters of particular interest because they impact both the distribution of suitable vector habitats and the potential for local vector proliferation^16^. Study of the relationships between temperature, the temporal patterns of dengue fever, and *Ae. aegypti* populations is common, while the relationship between spatial patterns, temperature, and *Ae. aegypti* remains poorly understood. Studies of urban micro-climate show that temperature can vary significantly over relatively short distances^23^, which are likely to impact mosquito populations^17^ and their capacity as vectors of disease^24–26^.

**Figure 1:**
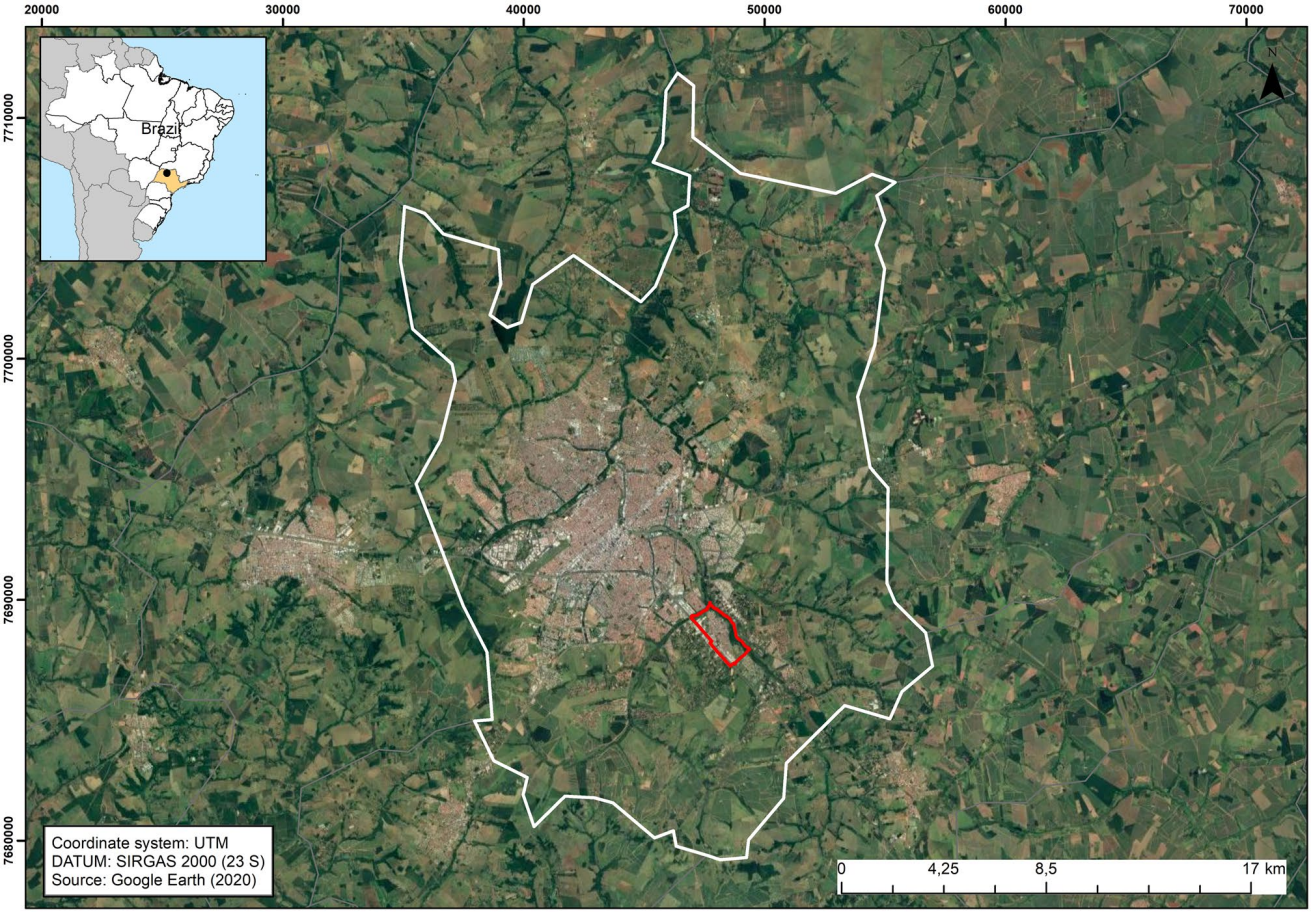
Municipality of São José do Rio Preto, state of São Paulo, Brazil. Vila Toninho neighborhood (study area) is highlighted in red. Map data: Google, Maxar Technologies.

Thermal satellite images can be used as input data for modelling mosquito infestation. For example, the Landsat-8 Thermal Infrared Sensor (TIRS) provides images at a relatively fine temporal (every two weeks) and spatial (30 m) scales. These datasets offer opportunities to improve the accuracy and precision of mosquito infestation prediction models. Most of the existing studies that have focused on the *Aedes* genus of mosquitos have employed satellite-derived surface temperature data^17,27,28^, which may differ from air temperature by several degrees^27^, especially during the day. The correlation between ground and air temperatures is, nevertheless, generally strong, and positive spatial correlations between surface temperature and disease vectors have been reported^29^. Therefore, surface temperature offers a measure that can be used to characterize the spatial relationships between *Ae. aegypti* infestation and temperature. Here, we sought to demonstrate the application of remote sensing technology for the prediction of mosquito infestation in the Vila Toninho neighborhood of São José do Rio Preto, São Paulo, Brazil (Fig. 1).

## Results

### Landscape and thermal features

**Figure 2:**
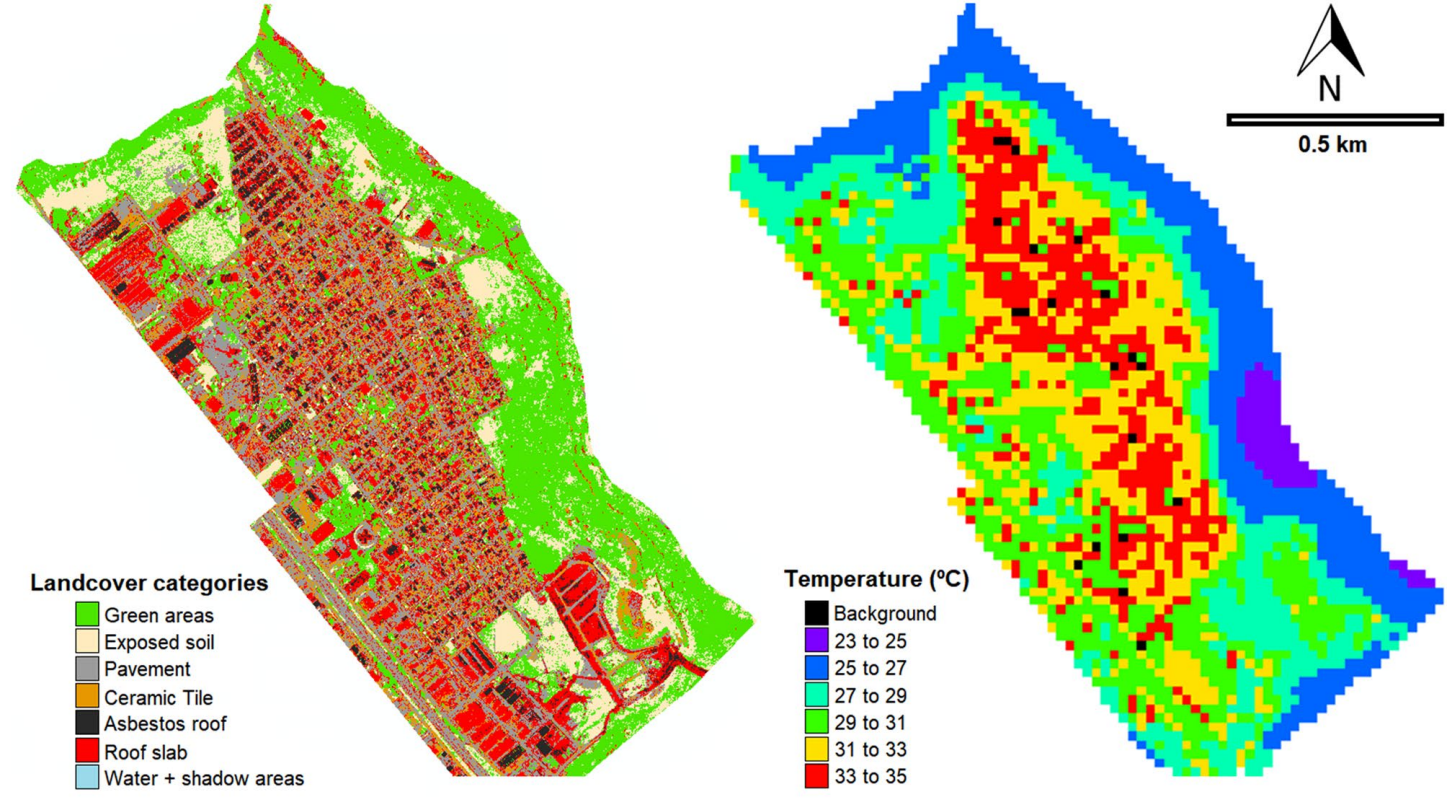
Vila Toninho neighborhood. Left: WorldView 3 image showing seven different landcover categories. Right: Landsat 8 TIRS showing surface temperature.

Supervised classification of landcover types based on WorldView-3 images after ground-truthing revealed that the most prevalent categories in the urban Vila Toninho neighborhood are pavement, ceramic tile, and roof slab (Fig. 2). These categories are also the most common within the 30 m buffers, those used to calculate mosquito infestation. This classification procedure had an overall estimation of 90.6% accuracy and a Kappa index (see “Methods” section) of 0.89. Classification errors occurred either as a result of overestimation (false positive) or underestimation (false negative). The class-specific producer’s accuracies ranged between 43% (water) and 96% (pavement) and the user’s accuracies between 57% (shadow areas) and 92% (ceramic tile). Water and shadowed areas yielded high percentages for both types of misclassification and were subsequently grouped under the single category ‘water + shadow areas’ during the statistical analysis.

Six different temperature zones (in increments of 2 °C, between 23 and 35 °C) were identified throughout the study area. These zones always adhered to the same spatial pattern, independently of season. Specifically, areas with high vegetation cover were cooler (25 ± 2 °C) than areas with moderate or low vegetation cover (both 31 ± 2 °C).

### *Aedes aegypti* infestation and multilevel modelling

**Figure 3:**
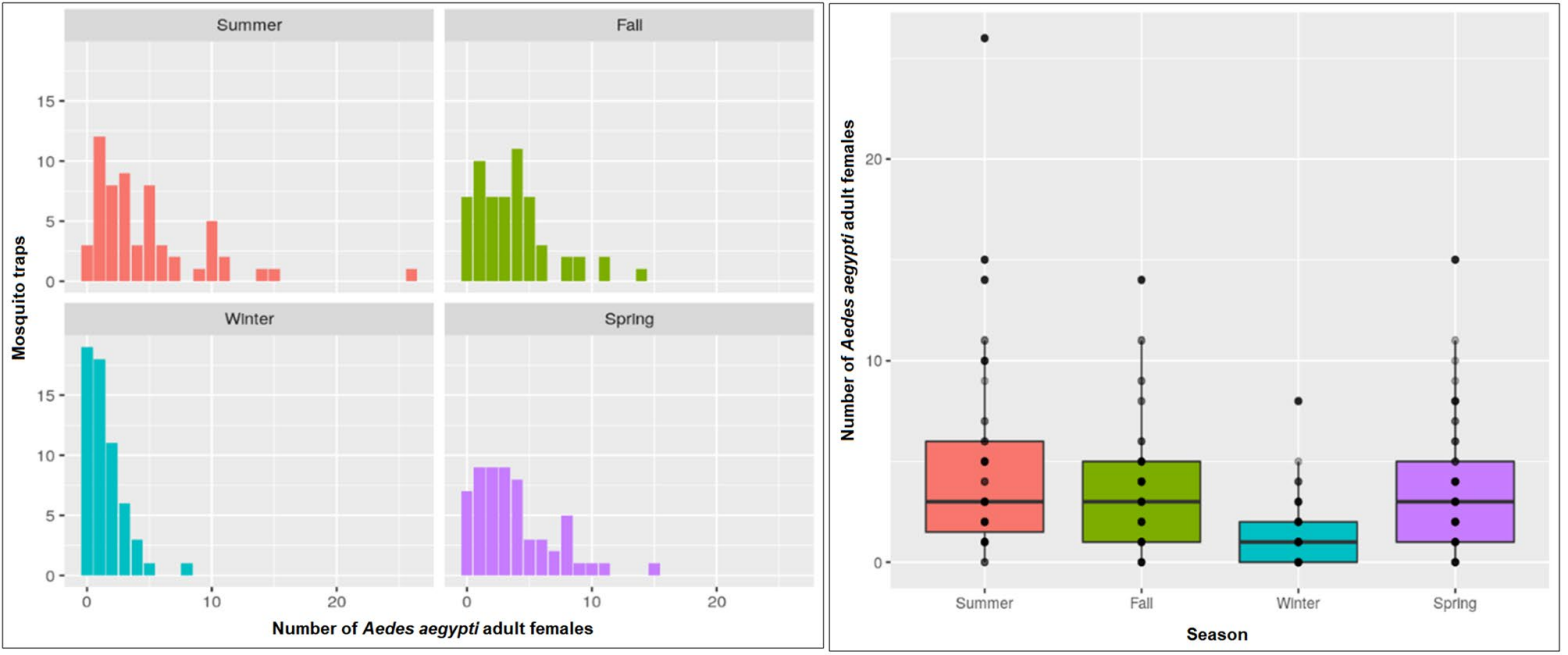
Distribution of *Ae. aegypti* adult female mosquitoes by season in Vila Toninho from 2016 to 2018.

During a 3-year monitoring period, we captured 788 *Ae. aegypti* adult females. The temporal variation in the observed infestation level is represented in Fig. 3, which indicates different levels of abundance during different seasons. Summer, fall, winter and spring presented a mean of 4.66, 3.59, 1.40, and 3.69 mosquitoes captured per trap, respectively. Notably, the lowest abundances occurred during the winter season (*p* < 0.001).

**Figure 4:**
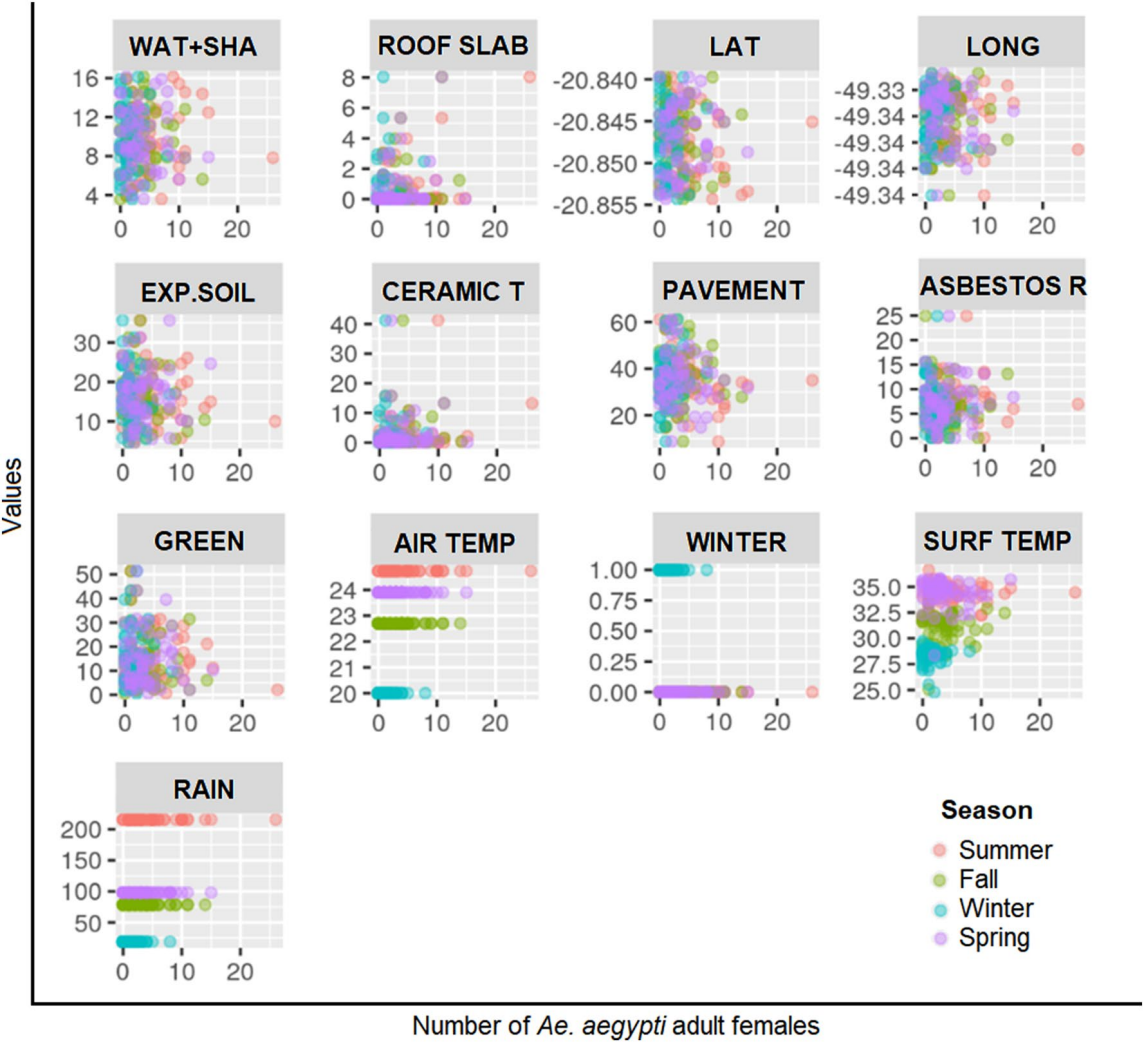
Scatterplots of each independent variable tested with the number of mosquitoes considering each season. Landcover categories (in %): *WAT + SHA* water and shadow, *EXP.SOIL* exposed soil, *CERAMIC T* ceramic tile, *ASBESTOS R* asbestos roof, *GREEN* green areas (trees and grass), *AIR TEMP* average season temperature (ºC), *WINTER* binary variable indicating if is winter season or not, *SURF TEMP* surface temperature (TIRS Landsat 8, ºC), *RAIN* average season rainfall (mm).

Exploratory analysis (Fig. 4) showed that latitude and longitude had no linear association with the number of mosquitoes (R^2^ =  − 0.0636 and − 0.0611, respectively). Collinearity analyses were performed, but no considerable relationships were observed. Average rainfall and average seasonal temperature were categorical variables that distinguished each season. The surface temperatures recorded near the mosquito traps were positively associated with the number of mosquitoes caught, which also varied between seasons. However, overall, the variable ‘season’ provided a suitable level of predictive power in the model so that surface temperature was subsequently discounted.

To explain the number of trapped *Ae. aegypti*, we used a multilevel binomial negative model in which the subject of the random variable was trap identification and incorporated information from repeated measures. The final model was produced by adjusting the inclusion and exclusion of explanatory variables from the complete model to achieve a satisfactory level of prediction. For example, latitude and longitude were excluded as they had no significant influence as exploratory variables. The cover types, ‘roof slab’ and ‘water and shadow areas’ were also excluded based on their lower statistical correlation with mosquito abundance. As the summer, autumn, and spring seasons did not show any difference in the model regarding mosquito abundance, we subsequently chose to represent season as a binary variable (i.e., ‘winter’ and ‘not winter’). Based on this, the following negative binomial model was derived:

$${\text{NUM}}\_{\text{AEDES }}\sim {\text{ Green }} + {\text{ Asbestos R }} + {\text{ Ceramic T }} + {\text{ Ex}}{\text{. soil }} + {\text{ PAV }} + {\text{ WINTER }} + \, \left( { \, 1|{\text{TRAP}}\_{\text{ID}}} \right)$$

where NUM_AEDES is number of adult *Ae. aegypti* females; Green is green areas; Asbestos R is asbestos roof; Ceramic T is ceramic tile; Ex. soil is exposed soil; PAV is pavement; WINTER identifies the season as winter (or otherwise); and TRAP_ID is the identifying number of each mosquito trap. Table 1 shows the coefficients for each of the variables selected to compose the model.

**Table 1:** Estimates of model parameters, standard error, and *p*-values.

Variable	Estimate	Std. error	Pr( >|z|)	Exp_Estimate
(Intercept)	4.7143817	0.9535441	0.0000008	111.5398228
Green areas	** − 0.0388395**	**0.0123143**	**0.0016104**	**0.9619051**
Asbestos roof	*0.0270723*	*0.0159589*	*0.0018139*	*1.0732909*
Ceramic tile	− 0.0445740	0.0219653	0.0524288	0.9564048
Exposed soil	*0.0513356*	*0.0149664*	*0.0006035*	*1.0499598*
Pavement	** − 0.0457916**	**0.0131612**	**0.0005027**	**0.9552410**
Winter	** − 1.0052431**	**0.1455796**	**0.0000000**	**0.3659557**

The variables in bold showed a negative association with the number of mosquitoes, while those in italics showed a positive association.

**Figure 5:**
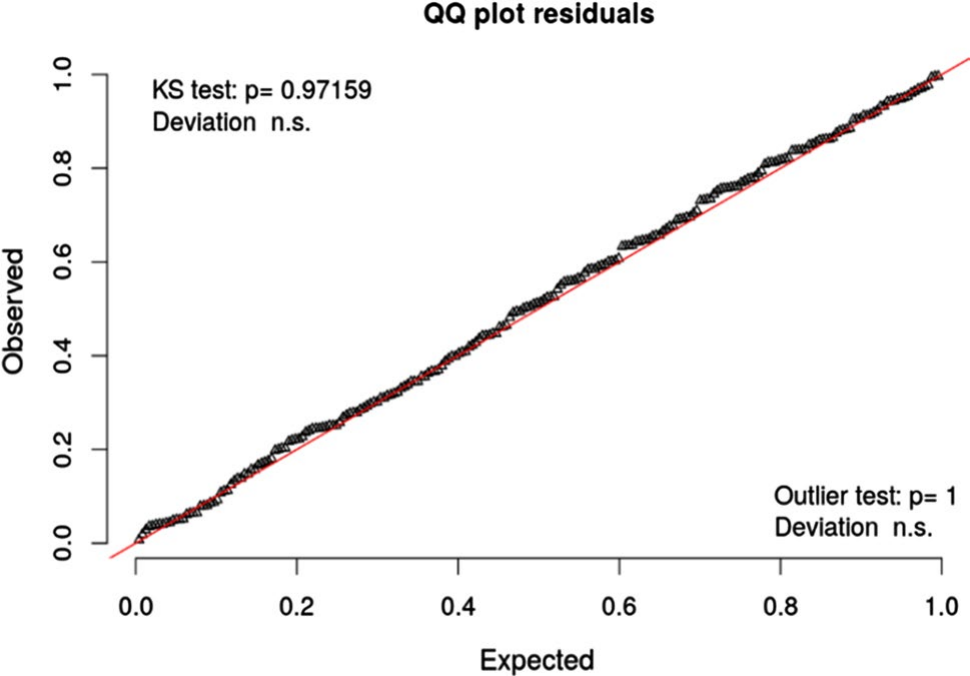
QQ-plot of the selected multilevel negative binomial model. *n.s* non-significant. The KS test indicates that the points are not far from the reference line (non-significant *p*-value). Similarly, the Outlier test did not reveal any significant presence of outliers in the data. It tests if the residues (expected/observed) have normal distribution and if there is any discrepant point.

The cover types ‘asbestos roof’ and ‘exposed soil’ had a positive association with the number of female mosquitoes, while ‘green area’, ‘paving’, and ‘winter’ were negatively associated with mosquito abundance. To evaluate the model fit, we first checked the model against the null model using Analysis of Variance (ANOVA). This showed that the negative binomial model performed significantly better than the null model (*p* < 0.0001), with an Akaike Information Criterion (AIC) of 1,047.42 compared to 1208.24, respectively (see Supplementary Material 1). We also simulated the QQ-plot residuals of our model according to Hartig^30^ (Fig. 5), which indicated that the model was suitable (see “Methods” section). Considering statistical significance, the KS test indicates that the points are not far from the reference line (non-significant p-value). Similarly, the Outlier test did not reveal any significant presence of outliers in the data. It tests if the residues (expected/observed) have normal distribution and if there is any discrepant point.

We calculated the root mean square error (RMSE) and the mean absolute error (MAE). The smaller the RMSE and MAE values, the better the model’s performance. As a comparison criterion, we considered the relative RMSE (Rel_RMSE) and relative MAE (Rel_MAE) as the error divided by the average of the observed response. This measure is similar to the variation coefficient. We obtained Rel_RMSE = 0.093 and Rel_MAE = 0.03, which indicates that the error is about 9% of the average considering RMSE and 3% of the average considering MAE. Generally, it is desirable to have a coefficient of variation less than 15% to prove the model’s effectiveness^31^. To validate our model, we used another dataset (summer of 2019) and also obtained the relative RMSE (0.1367) and relative MAE (0.0724). Although the values are higher, they still consider the model adequate.

**Figure 6:**
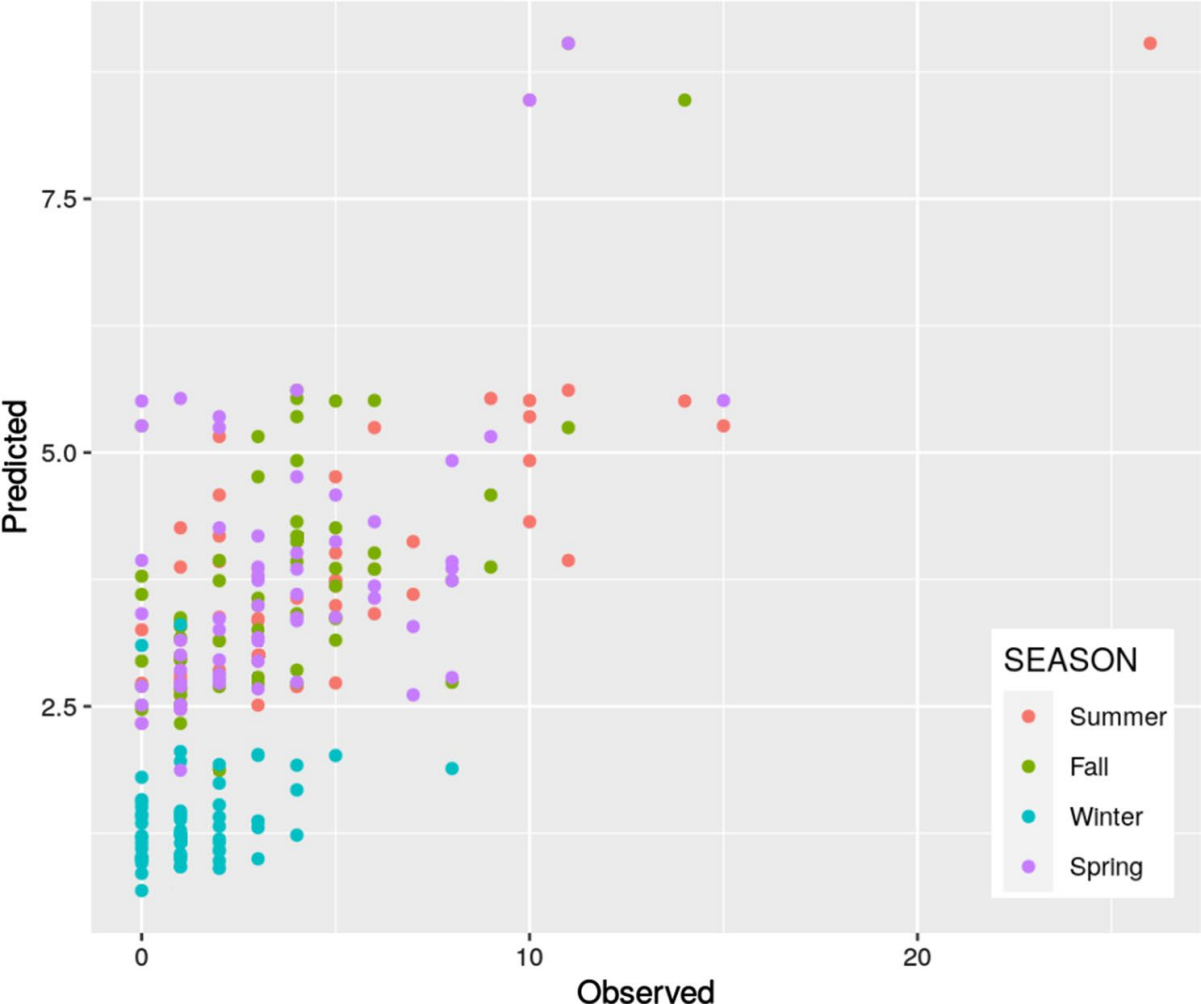
Number of *Ae. aegypti* adult females observed and predicted per trap using the negative binomial model pooled by season (correlation coefficient R = 0.74).

Figure 6 shows observed mosquito abundance against predicted abundance using our model (correlation coefficient R = 0.74). This shows that the model successfully captures the general pattern of the observations and helps to explain the seasonal variations in mosquito abundance observed in Vila Toninho.

## Discussion

Our findings indicate the potential for using remote sensing data in predictive models of *Ae. aegypti* infestation and other possible applications, such as dengue fever outbreak prediction^16,32^. Land-surface temperature data should be applied carefully as it is strongly correlated with season, precipitation, and air temperature. We observed that green (vegetated) areas always experience lower land-surface temperatures irrespective of season. Indeed, vegetation plays an essential role in controlling temperature fluctuations in urban settings, and urban expansion without the inclusion of vegetation is associated with significant temperature increases due to heat island effects^28^. Vegetation also affects the distribution of *Ae. aegypti* depending on other urban features, and independently of the surrounding geographical region^5^. For example, Hayden et al.^33^ used oviposition traps to evaluate the importance of microclimate and human factors in *Ae. aegypti* distributions in an arid environment of southwest United States and northwest Mexico. They found that mosquito eggs presence was positively associated with highly vegetated areas. Contrastingly in our study, levels of infestation were not associated with tree-covered areas. Although Vila Toninho is located in a tropical region^34^ with optimal conditions for the mosquito life cycle, the typically thick vegetation that grows there is not necessarily favorable for mosquitoes that prefer to breed within and around urban structures^5^. Similarly, natural water bodies, such as the rivers around Vila Toninho, do not serve as breeding sites for urban mosquito species that prefer artificial water containers for oviposition. Several studies have used land-surface temperature to model the preferred habitat conditions of other mosquito vectors, such as *Culex*^21^ and *Anopheles*^15^. However, this is a difficult task for *Ae. aegypti* because its breeding sites are small and preferentially distributed throughout the urban environment.

Our study is one of the first to apply infestation data specifically for adult females. Indeed, the adult population of *Ae. aegypti* is rarely sampled, due partly to the erroneous but commonly held belief that such sampling is time-consuming, difficult, or expensive^35^. For this reason, the vast majority of studies have focused on immature forms of the mosquito, but this may not be the best strategy for estimating disease risk since females possess additional epidemiological importance^35,36^. Getis et al.^37^ studied the spatial distribution of adult mosquito populations and found that they remain close to breeding sites, most at distances of approximately 10 m, up to 30 m, which can incorporate neighboring houses. Likewise, McDonald^38^ noted that adult *Ae. aegypti* spread to less than 20 m, and the majority of those recaptured after release were collected in the same house. Similarly, in Puerto Rico, Edman et al.^39^ collected most of their recaptured *Ae. aegypti* from the houses in which they were released. Whilst these studies indicate that in urbanized areas, such Vila Toninho, most adult *Ae. aegypti* do not fly far from the breeding sites in which they developed (typically inside households), the relationship between larval indices and adult densities is weakened by variable survival rates of immature forms and productivity by container type^40^.

The winter season was judged as the most influential factor in the decrease of mosquito infestations. São José do Rio Preto has a typically dry and cold winter (~ 19 °C) compared to other times of the year^41^. All other seasons have a similar average temperature (~ 24 °C) and levels of precipitation. Honório et al.^42^ found a positive linear relationship between *Ae. aegypti* infestation and air temperature within the range of 18 °C to 24 °C. These authors did not identify any variation in the abundance of mosquitoes above this temperature threshold. Furthermore, Eisen et al.^43^ showed a positive linear relationship between water temperature and the developmental rate of immature *Ae. aegypti* between 15 and 30 °C. Temperatures between 20 and 31 °C can increase the metabolic rate of mosquitos, shorten the period of larval development, and optimize foraging and egg-laying behavior leading to higher mosquito abundance when suitable larval habitats are available^26,28,29,44^. Despite reductions in the levels of infestation during winter, sufficient numbers of mosquitoes remain present to maintain the residual population until favorable weather conditions occur the following spring.

Despite that seasonal weather fluctuations can largely justify intra-annual fluctuations in the infestation levels of *Ae. aegypti*, several studies performed at the local scale and, consequently, under similar weather conditions, have revealed differences in mosquito abundance among contiguous urban areas^45–47^. This presumably reveals the effect of anthropogenic alterations in the urban environments that this mosquito prefers to inhabit, such as asbestos roofs and exposed soil, as was found in the current study. It is well accepted that, with the exception of the sylvatic ancestral form, *Ae. aegypti* mosquitoes are extremely anthropophilic, meaning that human settlements offer favorable conditions for the completion of their life cycle, especially in domestic settings^48^. In this sense, it is expected that environmental variables related to areas with buildings are good proxies for the abundance and distribution of *Ae. aegypti*, as demonstrated in previous remote sensing-based studies^14,49,50^. Our results have several similarities with Lorenz et al.^22^, as both studies showed that asbestos roof category was positively correlated with mosquito infestation, even using different types of statistical modelling and different periods of the year. Here we have included thermal features and could observe how the seasonality effect drastically affects the *Ae. aegypti* infestation.

Our multilevel modelling revealed that green areas and pavement cover are negatively associated with the presence of adult females, while areas with a higher percentage cover of asbestos roofs and exposed soil are positively associated with adult females. These variables reflect socio-economic conditions and also indicate differences in thermal capacity (particularly green areas) and the likelihood of surface water-ponding to create favorable breeding sites. Thus, green space expansion should be prioritized in urban planning, which may help reduce *Ae. aegypti* infestations if combined with maintained attempts to eliminate mosquito breeding sites and increase public comprehension of arbovirus transmission. Asbestos roofs are an inexpensive form of construction popular in poorer areas in Brazil^51^. The positive association between economically poor areas and mosquito infestation supports the findings of previous research^22,52–54^. In addition to affecting mosquito infestation levels, socioeconomic factors can be an important issue in the susceptibility of human inhabitants to arboviruses infection. For example, Hagenlocher et al.^55^ formulated an index of socioeconomic vulnerability to dengue that included both indicators of susceptibility, as well as a lack of resilience. The presence of paved areas prevents the accumulation of water as temporary breeding sites, unlike exposed soil. This finding is encouraging for the use of remote sensing to identify areas most at risk of high mosquito abundance at a local scale in urban settings. Successful prediction of the spatial distribution of suitable breeding habitats for *Ae. aegypti* would allow vector control efforts to target adult females more precisely (which have the greatest epidemiological importance), thereby reducing operational costs^56^.

The strengths of this study include the three-year monitoring period, the specific focus on adult female mosquitoes, and the use of high-resolution satellite images that allow the precise categorization of land cover. There are, however, some limitations. First, thermal satellite images do not have a sufficiently fine resolution to detect house-to-house-level variability. Data from the Landsat-8 TIRS have a spatial resolution of 100 m and a resampling resolution of 30 m. Second, we assumed that 30 m was the average flight radius of an adult female *Ae. aegypti*, but this may be an underestimate, depending on environmental conditions.

It is tempting to speculate that if remote-sensing approaches are effective at the city scale, characterized by heterogeneous landscape features, they might be even more effective in more homogeneous landscapes. In addition, it would be advantageous to develop further models using other freely available satellite images, including global datasets provided by organizations such as the United States National Aeronautics and Space Administration (NASA; https://lpcsexplorer.cr.usgs.gov/). Although the resolution of these datasets is not as high as Landsat-8, they might further support our observed patterns. In the future, health and surveillance workers should also consult free Google Earth images, for example, and locate high-risk areas based on the occurrence of certain land-cover types, including asbestos roofs and exposed areas of soil. It is worth mentioning that each region has a specific behavior concerning landcover type and mosquitoes, therefore they need specific plans for surveillance and control. For example, asbestos roofs may not be a good indicator of low socioeconomic levels in all countries, but in Brazilian areas they probably are. Improving our understanding of the linkages between landscape features and climatic variables, the incidence and spatial dissemination of mosquito vectors and arboviruses, and the capacity to use remotely-sensed information to recognize conditions signaling higher levels of risk would be of great value for optimizing vector control strategies, mosquito suppression activities, and outbreak prediction.

## Conclusions

We used remotely sensed temperature data and land-cover classification to identify features associated with adult female *Ae. aegypti* mosquitos in an urban neighborhood of São José do Rio Preto, São Paulo, Brazil. Reductions in mosquito infestations were most strongly associated with the winter season. In addition, green (vegetated) areas and pavements were negatively associated with the presence of adult females, while areas with a higher percentage cover of asbestos roofs and exposed soil were positively associated with female adults. These variables reflect local-scale socio-economic conditions but exhibit different thermal and water-ponding characteristics that offer more or less favorable breeding sites.

Our results have important implications for *Ae. aegypti* mosquito control in Brazil. Specifically, we have provided evidence that physical landscape characteristics influence the distribution of adult female mosquitoes. The local habitat aspects that control the mosquito life cycle often differ at spatial scales significantly finer than the land cover and census tract boundaries that inform most socio-environmental variables. As such, future studies should take into account observations of microhabitat characteristics that may affect the suitability of potential habitats for *Ae. aegypti*, sustained longitudinal entomological surveys including adult mosquito traps, and the incorporation of sociodemographic explanatory variables. Further work is now needed to analyze the identified associations over larger areas and in different socio-economic contexts.

## Methods

### Study site

This study was conducted in a neighborhood of the municipality of São José do Rio Preto in the state of São Paulo, Brazil^22,57^. *Aedes aegypti* mosquitos were reintroduced into the municipality in 1985^58^ and the first autochthonous case of dengue fever was confirmed in 1990. The study neighborhood, Vila Toninho (Fig. 1), is mainly urban and is located in the southeastern part of São José do Rio Preto with approximately 5,600 inhabitants (density = 4800 per km^2^)^34^ and 1,940 residences. Located on the outskirts of São José do Rio Preto, Vila Toninho has poorer socio-economic indicators than the city averages. The average income of the heads of households is 1.9 Brazilian minimum salaries (MS), and 15.3% of households have five or more residents. Comparative values for the entire municipality are 5.7 MS, and 11.5%, respectively^34^. The study area has undulating terrain and is characterized by dry winters with moderate temperatures and wet summers with moderately high temperatures^59^.

### Field survey

The procedures followed in our study were based on Lorenz et al.^22^. Adult mosquitoes were captured using 30 BG Mosquitito traps (Biogents BGS) installed in 2016 between December and February (the peak period of *Aedes* infestation) and monitoring continued until 2019. We used data from 2016 to 2018 to build the model and data from summer of 2019 to validate it. Traps were positioned near plant pots and out of direct exposure to sun and rain at preselected residences with shaded areas. New traps were installed twice a week, once per month, and at the same households, allowing us to gather data from up to 60 households each week. Traps were installed on Mondays and Thursdays and collected on the respective Tuesdays and Fridays (i.e. each trap was left in place for 24 h). The Cartesian coordinates of these houses and individual traps (Datum WGS-84, SIRGAS 2000) were obtained using a Global Positioning System (GPS). Mosquitos collected from the traps were identified at the Laboratory of Vectors, Medical School of São José do Rio Preto (FAMERP), based on taxonomic keys^60,61^. We focused on adult female mosquitoes given their epidemiological importance.

### Environmental data

Average precipitation and air temperature data for each season were obtained in ASCII-raster format and ‘LAT/LONG’ geodetic coordinate information (Datum WGS-84) from the WorldClim Global Climate Data database (https://www.worldclim.org/). These datasets contained observational data for 2016–2018, which were interpolated to a resolution of 30 arc-seconds (approximately 1 km). The precipitation and temperature data were used in the determination of the winter, summer, spring, and autumn seasons.

Land surface temperature was calculated using thermal remote sensing images from the Landsat-8 TIRS sensor, which are freely available from NASA’s website (https://lpcsexplorer.cr.usgs.gov/), offering a resolution of 100 m (resampled every 30 m) for Vila Toninho. Thermal band 10 was used to calculate land surface temperatures in the selected images. We used 18 satellite images obtained between 2016 and 2018 (Supplementary Material 2) representing all seasons of the three-year study period. Certain atmospheric conditions, such as haze and high humidity, resulted in pixel saturation and noise in the thermal imagery. Thus, images with saturated pixels or cloud cover were excluded. The Geographical Information System software, Qgis 2.14, was employed to estimate surface temperatures following Ndossi and Avdan^62^. Apparent temperatures were transformed from the digital signal of the satellite into radiance (w/m^2^·sr·μm). The digital number of each pixel was converted into monochromatic spectral radiance. Surface temperature was calculated using Planck’s inverted function^63^ from images of brightness temperature (band 10) and surface emissivity. The emissivity images were calculated based on the values of the vegetation index by normalized difference (NDVI) according to Valor and Caselles^64^, and Zhang et al.^65^. Information about air temperature and relative humidity^66^ on the specific capture days was also obtained to make corrections according to the weather conditions. Thus, it was possible to obtain surface temperature images representative of each season for Vila Toninho at a spatial resolution of 30 m.

With the images acquired and classified according to surface temperature for each season of the year, 30 m buffer zones were applied around each mosquito trap for which the average surface temperature was calculated (as a weighted average according to the corresponding area within the buffer). This gave a unique surface temperature value corresponding to each trap during each season. We were then able to correlate the number of *Ae. aegypti* females caught in each trap with the corresponding temperature.

### Landscape feature data

The procedures followed in our study were based on those of Lorenz et al.^22^. Cloud-free images of the study area were obtained from the WorldView-3 satellite (0.31 m in panchromatic mode and 1.24 m in the multispectral mode, resampled accordingly) acquired in March 2017. These datasets are composed of one panchromatic band (450–800 nm) and four multispectral bands comprising blue (450–510 nm), green (510–580 nm), red (630–690 nm), and near-infrared (770–895 nm). The supervised classification of images was performed using ArcGIS 10.5 by applying the Maximum Likelihood algorithm. These classifiers assigned each pixel to the following eight predetermined land-cover classes: (1) pavement; (2) tile roof; (3) asbestos roof; (4) roof slab; (5) green area; (6) exposed soil; (7) water; and (8) shadow areas. Classes 7 (water) and 8 (shadow areas) were subsequently combined into one class. We manually selected training samples (50 polygons per class) and test samples (50 polygons per class) corresponding to these eight categories. The classification accuracy was quantitatively assessed using the test samples, a confusion matrix, and the Kappa coefficient. The overall user and producer accuracies were also defined to evaluate the classification accuracy^67^. The overall accuracy is the ratio between all validation pixels correctly classified (the total correct pixels of each polygon) and validation pixels (the total number of pixels in the error matrix), whereas the user’s accuracy includes commission errors and the producer’s accuracy includes omission errors related to the individual classes^67^. The Kappa coefficient is a statistical measure of agreement that considered all of the categories. Values close to zero indicate that the observed agreement is the same as would be expected by chance and values approaching one indicate perfect agreement^68^.

Around each of the 60 traps, 30 m buffer zones were constructed, representing the assumed mean distance travelled by an *Ae. aegypti* female mosquito^36,69^. A study by Getis et al.^37^ showed that *Ae. aegypti* adults gathered strongly within houses close to breeding sites but were weakly clustered at a distance of 30 m beyond the household. We calculated the percentage of each land cover category in each buffer zone and compared these data to the number of *Ae. aegypti* adult females found in each trap.

### Data analysis

For multilevel modelling, the response variable was the number of female *Ae. aegypti* (indicating the level of infestation). The final predictive model was selected based on a comparison of Akaike Information Criterion (AIC) values for alternative distributions, including negative binomial, Poisson, and zero-inflated Poisson. To explain the number of *Ae. aegypti* mosquitoes, we used a mixed negative binomial model in which the subject of the random effect was the identification of the trap, and which incorporated repeated measures information (four seasons × three years). A negative binomial model was selected based on the performance of the model and the nature of the variables, which did not meet the assumption of equal mean and variance in the Poisson model.

The final model was derived by excluding those variables with the lowest significance until a model with all significant variables and acceptable goodness of fit was obtained. The goodness of fit was verified in three ways. First, the model was compared to the null model, i.e., a model with no independent variable, to verify that the addition of covariates was relevant for explaining the response variable. This comparison was made via ANOVA. Second, the model fit was verified visually by graphically comparing the observed data and the model predictions. Third, in our model we used data from 2016 to 2018, and we validated it using a new data set collected in the summer of 2019 with 60 observations (mosquito traps). We calculated the RMSE and MAE, both are regularly employed in model evaluation accuracy^70^. Finally, we used Hartig’s residual analysis^30^, in which a suitable model is expected to have residues located around the reference line of a simulated QQ-plot. KS test and Outlier test were also performed to check if the points are far from the reference line and if there are outliers in the sample, respectively. All analyses were performed using R version 3.6.1.

### Ethics

This study was approved by the Internal Review Board from the Medical School of São José do Rio Preto (FAMERP) (protocol #02078812.8.0000.5415). Homeowners who had traps installed on their properties signed an informed consent form.

## Supplementary Information

Supplementary Information 1.

Supplementary Information 2.

## Data Availability

The datasets generated during and/or analyzed during the current study are available from the corresponding author on reasonable request.
