# Supplementary material for: Predicting Aedes aegypti infestation using landscape and thermal features
Source: Sci Rep. 2020 Dec 10;10:21688. doi: 10.1038/s41598-020-78755-8 (PMC7729962; doi:10.1038/s41598-020-78755-8)
Supplement: Supplementary file 1 — Supplementary Information 1. [file 41598_2020_78755_MOESM1_ESM.pdf]

Predicting *Aedes aegypti* infestation using landscape and thermal features

Camila Lorenz[1*], Marcia C. Castro[2], Patricia Michele Pereira Trindade[3], Maurício Lacerda Nogueira[4], Mariana de Oliveira Lage[5], José Alberto Quintanilha[5], Maisa Carla Parra[4], Margareth Regina Dibo[6], Eliane Aparecida Fávaro[5], Marluci Monteiro Guirado[7], and Francisco Chiaravalloti-Neto[1]

Supplementary Material 1

ANOVA table comparing the null model (fitNULLMixed), the Poisson model (fitPoi) and the selected Negative Binomial model (fitNB). Df = degrees of freedom; AIC = Akaike information criterion; BIC = Bayesian information criterion.

	Df	AIC	BIC	logLik	deviance	Chisq	Chi Df	Pr (>Chisq)
**fitNULLMixed**	2	1208.243	1215.171	-602.1215	1204.243	NA	NA	NA
**fitPoi**	8	1100.202	1127.913	-542.1012	1084.202	120.04063	6	0
**fitNB**	9	1047.427	1078.601	-514.7133	1029.427	54.77574	1	0
