# Supplementary material for: Predicting Aedes aegypti infestation using landscape and thermal features
Source: Sci Rep. 2020 Dec 10;10:21688. doi: 10.1038/s41598-020-78755-8 (PMC7729962; doi:10.1038/s41598-020-78755-8)
Supplement: Supplementary file 2 — Supplementary Information 2. [file 41598_2020_78755_MOESM2_ESM.pdf]

Predicting *Aedes aegypti* infestation using landscape and thermal features

Camila Lorenz[1]*, Marcia C. Castro[2], Patricia Michele Pereira Trindade[3], Maurício Lacerda Nogueira[4], Mariana de Oliveira Lage[5], José Alberto Quintanilha[5], Maisa Carla Parra[4], Margareth Regina Dibo[6], Eliane Aparecida Fávaro[4], Marluci Monteiro Guirado[7], and Francisco Chiaravalloti-Neto[1]

Supplementary Material 2

LandSat 8 images used in thermal analysis. Air temperature and relative humidity on the day of image capture were obtained from WorldClim (2019).

ID	Acquisition Date	Season	Temp (ºC)	Hum (%)
1. LC08_L1TP_221075_20181116_20181129	16/11/2018	Spring	26	73.25
2. LC08_L1TP_221075_20180812_20180828	12/08/2018	Winter	18.6	68
3. LC08_L1TP_221075_20180201_20180220	01/02/2018	Summer	24.5	67.25
4. LC08_L1TP_221075_20171113_20171122	13/11/2017	Spring	24.7	46.5
5. LC08_L1TP_221075_20170622_20170630	22/06/2017	Winter	20.8	61.75
6. LC08_L1TP_221074_20170419_20180528	19/04/2017	Fall	23.5	70
7. LC08_L1TP_221074_20170129_20170214	29/01/2017	Summer	25.1	71
8. LC08_L1TP_221074_20161009_20170320	09/10/2016	Spring	25.6	51
9. LC08_L1TP_221074_20160721_20170323	21/07/2016	Winter	20.1	61.25
10. LC08_L1TP_221074_20160416_20170326	16/04/2016	Fall	28	83
11. LC08_L1TP_221074_20161110_20170318	10/11/2016	Spring	24.1	60.5
12. LC08_L1TP_221074_20160523_20170321	23/05/2016	Fall	21.4	79
13. LC08_L1TP_221074_20171129_20171207	29/11/2017	Spring	25.5	83
14. LC08_L1TP_221074_20171012_20171024	12/10/2017	Spring	24	83.2
15. LC08_L1TP_221074_20170910_20170927	10/09/2017	Spring	25.7	55.25
16. LC08_L1TP_221075_20180428_20180911	28/04/2018	Fall	21.2	46
17. LC08_L1TP_221074_20180727_20180731	27/07/2018	Winter	21.8	45.75
18. LC08_L1TP_221074_20180625_20180704	25/06/2018	Winter	22	60
